# Determination and prediction of the digestible and metabolizable energy contents of corn germ meal in growing pigs

**DOI:** 10.5713/ajas.17.0891

**Published:** 2018-05-24

**Authors:** Meng Shi, Zhaoyu Liu, Hongliang Wang, Chuanxin Shi, Ling Liu, Junjun Wang, Defa Li, Shuai Zhang

**Affiliations:** 1 State Key Laboratory of Animal Nutrition, Ministry of Agriculture Feed Industry Centre, China Agricultural University, Beijing 100193, China; 2 College of Biological and Food, Shangqiu Normal University, Shangqiu 476000, China

**Keywords:** Corn Germ Meal, Digestible Energy, Metabolizable Energy, Pig, Prediction Equation

## Abstract

**Objective:**

This experiment was conducted to determine the chemical composition, digestible energy (DE) and metabolizable energy (ME) contents of corn germ meals (CGM) and to develop equations to predict the corresponding energy contents based on the chemical characteristics of individual CGM.

**Methods:**

Sixty-six barrows (initial body weight = 51.3±4.6 kg) were allotted to 11 diets including a basal diet and 10 CGM test diets in a completely randomized design. In the test diets, CGM was included in replacement of 30% of the energy-providing ingredients in the basal diet, resulting in a final inclusion rate of 29.1%. Each diet was fed to 6 barrows housed in individual metabolism crates for a 7-d acclimation period followed by a 5-d total but separate collection of feces and urine.

**Results:**

Considerable variation was observed in acid-hydrolyzed ether extract, ether extract, ash, calcium (Ca) and total phosphorus contents among the CGM samples. On dry matter (DM) basis, the DE and ME contents of the CGM ranged from 10.22 to 15.83 MJ/kg and from 9.94 to 15.43 MJ/kg, respectively. The acid detergent fiber (ADF) contents were negatively correlated with the DE and ME contents of CGM samples. The best-fit prediction equations for the DE and ME values (MJ/kg DM) of the 10 CGM were: DE = 26.85–0.28 insoluble dietary fiber (%)–17.79 Ca (%); ME = 21.05–0.43 ADF (%)–11.40 Ca (%).

**Conclusion:**

The chemical compositions of CGM vary depending on sources, particularly in ether extract and Ca. The DE and ME values of CGM can be predicted based on their chemical composition in growing pigs.

## INTRODUCTION

Corn germ meal (CGM) is produced during the process of germ extraction to produce corn oil for human consumption [1]. As a by-product of the oil industry, CGM could provide relative abundant protein and energy contents, which was firstly thought to be used in pet food to replace part of the more expensive corn and also a small portion of the soybean meal [2]. However, the high fiber content of CGM limits its utilization primarily to ruminant diets [3,4] and fish diets [2]. Recently, researchers began to put more attention on the application of CGM in swine diets. Soares et al [5] reported that the inclusion rate of CGM in diet for growing and finishing pigs could reach 30% without disturbing the economical parameters. Weber et al [6] showed that an inclusion level of 38% CGM in diets did not affect the average daily gain and average daily feed intake of pigs, but did reduce the feed efficiency. Nevertheless, few data are available on the energy contents of CGM fed to pigs, except that Anderson et al [7] presented a relatively detailed research on the digestible energy (DE) value of CGM in pigs, but with only one sample. The composition and quality of CGM can vary considerably [6,8,9] due to different processing conditions during oil extraction as well as different soil, latitude and environmental conditions during corn growing and harvesting [10]. As a result, it is necessary to evaluate the energy contents of CGM in pigs with more samples.

Precise estimation of DE and metabolizable energy (ME) values are essential for accurate diet formulation and feed cost reduction. However, it consumes large amount of labor, time and money through traditional *in vivo* metabolism experiments to measure the energy contents [11]. Instead, many researchers reported prediction equations to estimate the available energy based on the chemical compositions, for example, in the complete diets [12], distillers dried grains with soluble [13] and corn co-products [7]. No such equations have been generated by now on CGM in growing pigs. Therefore, we hypothesized that there are differences in DE and ME values of CGM samples because of their variable chemical composition. The objectives of the present study were to determine the chemical compositions and DE and ME values of CGMs obtained from different plants and to develop prediction equations to estimate the DE and ME values of individual CGM sample.

## MATERIALS AND METHODS

The Laboratory Animal Welfare and Animal Experimental Ethical Inspection Committee of China Agricultural University (Beijing, China) reviewed and approved all protocols used in this experiment.

### Source of ingredients

Ten samples of CGM were collected from 10 commercial wet-milling plants, including corn starch plants and oil plants, in the major CGM production area of China (Table 1). In order to make the samples representative, a survey was conducted and information including the scale of enterprise, source of raw materials and plant output were obtained before collecting the samples. All samples were stored at −18°C before chemical analysis and diet formulation. The CGM sub-samples were collected and analyzed for chemical compositions before the animal trial (Table 2).

### Animals and experimental design

Sixty-six healthy crossbred barrows (initial body weight [BW] of 51.3±4.6 kg, Duroc×Landrace×Yorkshire) were assigned to 11 treatment diets in a completely randomized design, with 6 replicate pigs per diet. All pigs were placed in individual stainless steel metabolism cages (1.4×0.45×0.6 m^3^) on concrete slatted floors and stayed in temperature-controlled rooms (22.0°C±1.3°C). The cages were equipped with a feeder in front and a dripper on one side, which could limit the feed spillage and keep the feed separate from water. The animal trial lasted for 12 d including 7 d for diet and cage adaptation followed by a 5-d period of total feces and urine collection.

### Diets and feeding

Pigs in the control group were fed a basal diet containing 76% corn, 21% soybean meal and 3% minerals and vitamins (Table 3). In the test diets, CGM was included in replacement of 30% of the energy-providing ingredients in the basal diet, resulting in a final inclusion level of 29.1%. Minerals and vitamins were supplemented in all diets to meet or exceed the estimated nutrient requirements for growing pigs recommended by NRC [14].

Barrows had free access to water and were fed an amount of daily feed equivalent to 4% of their BW determined at the beginning of the study [15]. The ration was divided equally into 2 feedings provided at 0800 and 1700. The amount of feed provided was recorded at each feeding. Feed refusals and spillage were collected twice daily and subsequently dried and weighed for daily feed consumption calculation.

### Sample collection

Samples of the diets and ingredients were collected and stored at −20°C until analysis. During the 5-d collection period, all feces were promptly collected into plastic bags and stored at −20°C according to the methods described by Song et al [16] and Ren et al [17]. At the end of collection period, the total 5-d production of feces from each pig was pooled and weighed, and a subsample of 300 g was taken and dried in a forced-draft oven at 65°C for 72 h. After drying and grinding, subsamples were stored at −20°C for further chemical analysis.

Urine samples were collected into plastic buckets attached to funnels located under the metabolic crates at the same time as fecal collection according to the methods described by Li et al [18]. The buckets (5 liters) contained 10 mL of 6 *N* HCl for every 1,000 mL of urine to limit microbial growth and reduce the loss of ammonia. The total urine volume excreted from each pig was recorded daily and 10% of the total volume was stored at −20°C. At the end of the collection period, the stored urine samples were pooled for each pig, and a subsample (about 4 to 5 mL) was saved for further analysis. The subsamples were dried at 65°C for 8 h with quantitative filter paper in crucibles for gross energy (GE) determination. Two unaltered sheets of quantitative filter paper from the same box were used to calibrate the energy content of the paper.

### Chemical analysis and calculations

The ingredients, diets and feces were analyzed for dry matter (DM, method 930.15), crude protein (CP, method 990.03), ash (method 975.03), calcium (Ca, method 985.01), and phosphorus (P, method 985.01) [19]. Acid-hydrolyzed ether extract (AEE) was determined by acid hydrolysis using 3 *N* HCl followed by ether extract (EE) extraction with petroleum ether (method 2003.06) [19] using Soxtec 2050 Automated Analyzer (FOSS North America, Eden Prairie, MN, USA). Neutral detergent fiber (NDF) and acid detergent fiber (ADF) were determined using fiber bags and fiber analyzer equipment (Fiber Analyzer, Ankom Technology, Macedon, NY, USA) following an adapted procedure described by van Soest et al [20]. Total dietary fiber (TDF) and insoluble dietary fiber (IDF) were determined according to the methods described by Prosky et al [21] and Prosky et al [22], respectively. Soluble dietary fiber (SDF) was calculated by subtracting IDF from TDF. Starch content was measured with the Ewers polarimetric method [23], but it was not measured in feces and the digestibility coefficient of starch was assumed to be 100%. The GE of feed, feces and urine were determined using an automatic isoperibol oxygen bomb calorimeter (Parr Instruments, Moline, IL, USA). Urine samples were analyzed in triplicate for GE value to gain an accurate result, and other samples were analyzed in duplicate.

The DE value in each diet was calculated as the difference between GE in diet and GE in feces. The ME value in each diet was calculated by subtracting the GE in urine from DE in diet. The energy content of the CGM in each diet was calculated using the difference method [15] by subtracting the DE and ME contributed by the other energy contributing ingredients in the basal diet.

### Statistical analysis

Data were checked for normality and equal variances using the UNIVARIATE procedure of SAS (Version 9.0; SAS Inst. Inc., Cary, NC, USA), and no outliers were found. All the data were analyzed by analysis of variance using the general linear model procedure of SAS with individual pig as the experimental unit. The statistical model included the treatment diet as fixed effect. Multiple comparisons were adjusted using Tukey’s method. The correlation coefficients among the proximate chemical composition and energy values (GE, DE, and ME) of CGM samples were calculated using the CORR procedure of SAS (USA).

Prediction equations for the DE and ME values of the CGM were developed using the REG procedure of SAS. Stepwise regression was used and chemical compositions were selected as prediction variables. Variables with p-values <0.05 were retained in the model. The R^2^, p-value and residual standard deviation were used to define the best-fit equation.

## RESULTS

### Chemical compositions of corn germ meal

As shown in Table 2, there was considerable variation in the proximate compositions of the 10 CGM samples. The coefficients of variation of all compositions except DM, CP, NDF, TDF, IDF, and GE were greater than 10%. The DM content of the 10 samples averaged 91.92%, with a range of 90.87% to 93.09%.

### Daily balance of gross energy

There were no differences in the daily feed intake, daily GE intake and daily GE loss in urine among pigs fed different CGM diets (Table 4). However, the daily feces output was lower (p = 0.02) for pigs fed CGM 1, CGM 3, CGM 5, and CGM 8 than those fed CGM 9, and the daily GE loss in feces was lower (p< 0.01) for pigs fed CGM 1, CGM 5, and CGM 8 than those fed CGM 9.

### Energy contents of corn germ meal diets and ingredients

The DE and ME values of the 10 experimental diets are demonstrated in Table 5. Diets supplemented with CGM 9 had the lowest (p<0.01) DE and ME values compared with the other experimental diets. Moreover, diets with CGM 1 added had greater (p<0.01) ME value compared to diets with CGM 4 added. No significant differences were observed for the ME/DE ratio among the 10 experimental diets. The DE and ME values, apparent total tract digestibility (ATTD) of GE and ME/DE ratios of the 10 CGM samples are presented in Table 5. The DE value and ATTD of GE in CGM 9 was lower (p<0.01) than those in the other CGM samples. The ME value in CGM 4 was higher (p<0.01) than that in CGM 9 but lower (p<0.01) than that in CGM 1. Otherwise, no significant differences were observed among the CGM samples for those parameters. The DE values of the CGM samples tested ranged from 10.22 to 15.83 MJ/kg on DM basis. The ME values of the CGM samples tested ranged from 9.94 to 15.43 MJ/kg on DM basis. There was no significant difference in the ME/DE ratio among different CGM samples.

### Correlation analysis

The correlation coefficients (*r*) between chemical compositions and the GE, DE, and ME values of the 10 CGM samples are shown in Table 6. Digestible energy and ME values were positively correlated with CP and starch (p<0.05), and were negatively correlated with ADF, IDF, and Ca (p<0.05). Gross energy had a negative correlation with ash, Ca and total phosphorus (p<0.05), and a positive correlation with AEE, EE, NDF, and TDF. The TDF was positively correlated with NDF, IDF, and SDF (p<0.05). Insoluble dietary fiber had a positive correlation with NDF and ADF (p<0.05). The highest correlation was found between DE and ME (*r* = 0.99, p<0.05).

### Prediction equations for DE and ME

Prediction equations were established to estimate the DE and ME values of CGM using regression analysis based on their chemical characteristics (Table 7). The ADF was the best predictor for both DE and ME values of the CGM. With the addition of Ca, the R^2^ of the prediction equations for the DE and ME values improved from 0.75 to 0.90 and 0.73 to 0.87, respectively. The R^2^ of the prediction equation for the DE was improved with the IDF instead of ADF. Moreover, DE and ME contents were highly correlated: ME (MJ/kg DM) = 0.40+0.93 DE (MJ/kg DM).

## DISCUSSION

### Chemical composition of corn germ meal

To establish the prediction equations for DE and ME values in CGM, 10 CGM samples with different sources were collected in this experiment. There was large variation among the chemical compositions of samples (Table 2). The chemical compositions were different from those in NRC [14] and previous literature [7]. The large variation existing in chemical characteristics such as AEE, EE, SDF, ash and Ca can be explained by the differences in original corn and the processing line. Soil, latitude and environmental conditions during corn growing and harvesting can play an important role in the changes of corn chemical components [10,24,25], resulting in different nutrient contents in CGM. The varied ash contents among the 10 CGM samples may be due to the corn source and the stone-removing step during the production process [10,24,25]. The varied fiber contents of CGM were related to the supplementation of corn bran to corn germ by the producer during processing [10,24,25]. Cooking temperature, solvent and machines used in the pre-press solvent extraction process would lead to variable fat residue in CGM [10,24,25]. To meet the customer requirements and to create more profit, some steep liquor was added to CGM, leading to the varied CP content of CGM obtained from different sources [3]. Among the components, the DM and GE values were relative stable.

### Energy content of corn germ meal

The averaged energy contents of the 10 CGM samples (14.42 MJ DE/kg DM and 13.78 MJ ME/kg DM, respectively) were comparable to those published by NRC (13.86 MJ DE/kg DM and 13.13 MJ ME/kg DM) [14]. The CGM of source 9 had the lowest DE and ME contents, which may be due to its lower GE and EE contents as well as higher ADF and IDF contents compared with other CGM samples. The fiber content of a feed ingredient is negatively correlated with its energy value [26]. Weber et al [6] showed that increasing dietary fiber content could decrease the absorption efficiency of dietary lipid and energy. The difference between the minimum and the maximum values of DE and ME contents were 5.61 and 5.49 MJ/kg among the 10 CGM samples on DM basis, respectively, which can result in considerable error when averaged energy values were used in diet formulation. Therefore, dynamic comparisons and prediction equations are necessary to accurately predict the energy contents of different CGM samples.

### Correlation analysis

Acid detergent fiber, a measurement of the total cellulose and lignin contents, was the most significant factor affecting the variation of the DE and ME contents in the dataset. As expected, there is a positive correlation between GE and AEE in CGM in our experiment because lipids provide more energy than carbohydrates and crude protein [27]. The components providing little energy, such as ash, calcium and total phosphorus, showed negative correlations with GE. Moreover, the concentration of fiber components such as ADF and IDF were negatively correlated with DE and ME in the current experiment, which is in agreement with the previous literature reporting negative influences of dietary fiber content on energy values [12,26]. However, it is difficult to explain why neither EE nor AEE had correlation with DE or ME.

### Prediction equations for DE and ME

Because of the large amount of labor, time and money required for a metabolism experiment, many researchers [7,12,28–31] take advantage of prediction equations to estimate DE and ME values in feed ingredients and diets. Noblet and Perez [12] presented that the DE and ME contents in swine diets could be accurately predicted from chemical components, using 114 diets with more than 40 ingredients. However, those prediction equations obtained may be more suitable for compound diets rather than an individual ingredient [18]. Because more similarities exist among different samples for a specific ingredient, more suitable prediction equations could be developed. Nevertheless, little research has focused on this work, and no specific prediction equations established solely targeting for the energy contents of CGM in pigs.

It is noteworthy that ADF instead of NDF was a suitable predictor in predicting DE and ME contents of CGM. A simple linear regression analysis of the current data suggests that for every 1% increase in ADF, there is a 3% (0.62 MJ) decrease in DE (Eq. 1, Table 7). The fact that IDF was selected into the best-fit prediction equation for DE indicates that the analysis of feedstuffs for TDF and IDF, especially in corn co-products is necessary. Inclusion of a second characteristic (Ca) into the equations improved the accuracy for DE and ME estimation. Generally, energy values are more related to organic compounds such as CP, EE, or starch [28]. Although the concentration of fiber (e.g., ADF) may be negatively related to energy values, the relationship between energy values and Ca concentration is hardly expected because the concentration of Ca in CGM is considerably lower compared with the other nutrients. Therefore, from the biological perspective, prediction equations with only ADF as the parameter (Eq. 1 and 4, Table 7) are more acceptable. However, the addition of parameter Ca greatly increased the R^2^ and decreased the residues of the equations, which means that it greatly improved the accuracy of prediction. Even though the Ca concentration is low, the coefficients of Ca in the prediction equations are large, which can ensure the logical prediction using Ca from the mathematical point of view. In addition, the processing condition of CGM from different production plants may affect the oil content of CGM, and the steep addition may affect the CP content of CGM, which are 2 important factors that could affect the energy content of CGM. As a result, adding these parameters, e.g. the processing conditions of each plant and the steep addition during the processing, into the equation may greatly improve the prediction efficiency. Nevertheless, this information is unavailable in the current experiment. Further investigation on processing conditions are needed in the future.

In conclusion, the ten CGM samples from different sources used in this experiment showed large variability in chemical characteristics, but no significant differences were observed in the energy contents for most tested samples. To get accurate prediction for the energy contents of the individual CGM sample, it would be conducive to develop prediction equations based on the chemical compositions of the CGM samples. The best-fit prediction equations on mathematical meanings for the DE and ME values (MJ/kg DM) of CGM were: DE = 26.85–0.28 IDF (%)–17.79 Ca (%); ME = 21.05–0.43 ADF (%)–11.40 Ca (%).

## Figures and Tables

**Table 1:** Sources of corn germ meals

No.	Province	Plant	Enterprise scale	Yield, thousand tons/yr
1	Shandong	Xi Wang Food Company	Large-sized private enterprise	300
2	Shandong	Liang You Trade Company	Small-sized private enterprise	48
3	Hebei	Ao Bang Oil Company	Small-sized private enterprise	26
4	Henan	Zhong He Starch Company	Small-sized private enterprise	28
5	Jilin	Zheng Wang Oil Company	Small-sized private enterprise	54
6	Shandong	San Xing Oil Company	Large-sized private enterprise	250
7	Shandong	Guang Yuan Corn Development Company	Small-sized private enterprise	38
8	Jilin	Tian Cheng Corn Development Company	Small-sized private enterprise	30
9	Hebei	Jin Dou Zi Oil Company	Small-sized private enterprise	30
10	Inner Mongolia	De Rui Corn Starch Company	Small-sized private enterprise	38

**Table 2:** Analyzed chemical compositions of corn germ meals used in the experiment (%, as-fed basis)1)

Item	Corn germ meal source2)										Mean	CV (%)

	1	2	3	4	5	6	7	8	9	10		
Dry matter	91.27	92.46	92.29	92.26	93.09	91.72	90.87	92.04	92.10	91.11	91.92	0.74
Crude protein	18.27	18.77	19.64	19.26	22.73	18.09	17.23	21.15	16.97	18.71	20.75	8.67
Acid-hydrolyzed ether extract	2.40	3.70	2.22	1.68	2.00	3.04	3.44	7.06	1.75	2.92	3.29	52.23
Ether extract	1.35	2.62	0.72	0.59	0.92	1.61	2.15	5.56	0.32	1.60	1.90	87.08
Neutral detergent fiber	47.29	45.78	44.93	47.98	37.88	50.71	49.69	45.03	43.67	50.48	50.44	8.88
Acid detergent fiber	13.34	12.42	12.64	13.14	10.10	13.41	14.49	11.33	17.40	12.92	14.28	14.96
Total dietary fiber	53.36	49.21	43.83	48.47	44.09	53.21	50.00	49.40	50.00	54.31	53.97	7.76
Insoluble dietary fiber	44.25	39.61	42.71	44.24	37.33	45.60	44.12	37.64	48.61	46.96	46.91	9.25
Soluble dietary fiber	9.11	9.60	1.12	4.23	6.75	7.61	5.88	11.77	1.39	7.35	7.05	53.12
Starch	14.42	16.48	16.71	17.43	12.38	13.93	15.35	13.31	9.88	15.95	15.87	15.89
Ash	1.69	4.35	2.20	2.48	4.98	1.51	2.64	2.66	4.59	1.42	3.10	45.67
Calcium	0.03	0.15	0.04	0.05	0.10	0.04	0.07	0.04	0.21	0.04	0.09	75.48
Total phosphorus	0.33	0.43	0.39	0.42	0.71	0.31	0.31	0.54	0.34	0.33	0.45	30.73
Gross energy (MJ/kg)	17.66	17.71	17.35	17.61	17.60	17.96	17.92	17.75	17.39	17.95	17.59	1.75

CV, coefficients of variation.

1) Analysis conducted in duplicate.

2) Sources of corn germ meal were detailed in Table 1.

**Table 3:** Ingredient compositions of the experimental diets (%, as-fed basis)

Item	Basal diet	Test diets
Corn	76.00	53.20
Soybean meal, 46% CP	21.00	14.70
Corn germ meal	-	29.10
Dicalcium phosphate	1.10	1.10
Limestone	0.90	0.90
Sodium chloride	0.30	0.30
Choline chloride	0.20	0.20
Mineral and vitamin premix1)	0.50	0.50

CP, crude protein.

1) Premix provided the following per kg of complete diet for growing pigs: vitamin A, 5,512 IU; vitamin D_3_, 2,200 IU; vitamin E, 64 IU; vitamin K_3_, 2.2 mg; vitamin B_12_, 27.6 μg; riboflavin, 5.5 mg; pantothenic acid, 13.8 mg; niacin, 30.3 mg; choline chloride, 551 mg; Mn, 40 mg (MnSO_4_); Fe, 100 mg (FeSO_4_·H_2_O); Zn, 100 mg (ZnSO_4_); Cu, 100 mg (CuSO_4_·5H_2_O); I, 0.3 mg (KI); Se, 0.3 mg (Na_2_SeO_3_).

**Table 4:** The daily feed intake, daily feces output and daily balance of GE for growing pigs fed corn germ meal diets1)

Item	Corn germ meal source										SEM	p-value

	1	2	3	4	5	6	7	8	9	10		
Daily feed intake (kg/d)	2.02	2.01	2.04	1.95	1.98	2.00	2.04	1.98	2.00	1.99	0.03	0.99
Daily feces output (kg/d)	0.99^b^	1.10^ab^	1.01^b^	1.14^ab^	0.92^b^	1.10^ab^	1.09^ab^	0.92^b^	1.26^a^	1.02b	0.02	0.02
Daily balance of GE (MJ/d)												
GE intake	37.48	36.85	37.57	36.00	36.31	36.99	37.71	36.62	36.63	36.95	1.50	0.10
GE in feces	5.17^b^	5.48^ab^	5.42^ab^	5.72^ab^	5.19^b^	5.66^ab^	5.90^ab^	5.06^b^	6.73^a^	5.72^ab^	0.29	<0.01
GE in urine	0.50	0.64	0.78	0.63	0.78	0.51	0.70	0.69	0.47	0.74	0.11	0.34

GE, gross energy; SEM, standard error of the mean.

1) Data are means of 6 observations per treatment.

a,b Within a row, different superscripts indicate a significant difference (p<0.05).

**Table 5:** Digestible energy (DE), metabolizable energy (ME), digestibility and metabolizability of gross energy (GE) and the ratio of ME to DE (ME/DE) of the 10 experimental diets, and DE, ME, apparent total tract digestibility (ATTD) of GE and the ratio of ME to DE (ME/DE) of the 10 corn germ meal samples fed to growing pigs1)

Item	Corn germ meal source										SEM	p-value

	1	2	3	4	5	6	7	8	9	10		
Diets												
DE (MJ/kg DM)	15.57^a^	15.24^a^	15.29^a^	15.02^a^	15.27^a^	15.20^a^	15.18^a^	15.42^a^	14.43^b^	15.19^a^	0.12	<0.01
ME (MJ/kg DM)	15.30^a^	14.88^ab^	14.87^ab^	14.66^b^^c^	14.84^ab^	14.91^ab^	14.80^ab^	15.04^ab^	14.17^c^	14.78^ab^	0.12	<0.01
ME/DE (%)	98.24	97.63	97.24	97.62	97.18	98.09	97.51	97.52	98.19	97.30	0.35	0.28
GE digestibility (%)	72.99^a^	65.74^a^	70.56^a^	64.68^a^	68.96^a^	66.10^a^	69.06^a^	71.42^a^	54.12^b^	65.77^a^	2.13	<0.01
GE metabolizability (%)	71.36^a^	62.62^ab^	66.26^a^	61.54^ab^	64.54^a^	64.22^a^	65.47^a^	67.88^a^	52.67^b^	61.80^ab^	2.13	<0.01
Corn germ meals												
DE (MJ/kg DM)	15.78^a^	14.31^a^	15.27^a^	14.28^a^	15.83^a^	14.26^a^	14.87^a^	14.52^a^	10.22^b^	14.87^a^	1.00	<0.01
ME (MJ/kg DM)	15.43^a^	13.90^ab^	14.52^ab^	13.42^b^	14.94^ab^	13.58^ab^	13.92^ab^	13.76^ab^	9.94^c^	14.35^ab^	1.00	<0.01
ATTD of GE (%)	72.99^a^	65.61^a^	70.55^a^	64.68^a^	68.96^a^	66.10^a^	65.99^a^	71.20^a^	54.12^b^	65.77^a^	5.17	<0.01
ME/DE (%)	97.74	95.30	93.81	95.25	93.70	97.14	94.81	95.00	97.41	94.13	3.34	0.31

SEM, standard error of the mean.

1) Data are means of 6 observations per treatment.

a,b Within a row, different superscripts indicate a significant difference (p<0.05).

**Table 6:** Correlation coefficients (*r*) between chemical constituents and energy values of the 10 corn germ meal samples1)

Item	DE	ME	GE	CP	AEE	EE	NDF	ADF	TDF	IDF	SDF	Starch	Ash	Ca
ME	0.99	1.00	-	-	-	-	-	-	-	-	-	-	-	-
GE	0.40	0.41	1.00	-	-	-	-	-	-	-	-	-	-	-
CP	0.57	0.54	−0.31	1.00	-	-	-	-	-	-	-	-	-	-
AEE	0.26	0.28	0.64	−0.27	1.00	-	-	-	-	-	-	-	-	-
EE	0.34	0.37	0.55	−0.16	0.97	1.00	-	-	-	-	-	-	-	-
NDF	0.04	0.05	0.84	−0.67	0.49	0.40	1.00	-	-	-	-	-	-	-
ADF	−0.80	−0.79	−0.01	−0.89	−0.09	−0.20	0.38	1.00	-	-	-	-	-	-
TDF	−0.12	−0.06	0.66	−0.61	0.41	0.38	0.77	0.42	1.00	-	-	-	-	-
IDF	−0.55	−0.53	0.39	−0.78	−0.08	−0.21	0.66	0.82	0.69	1.00	-	-	-	-
SDF	0.49	0.55	0.40	0.12	0.63	0.76	0.23	−0.43	0.50	−0.29	1.00	-	-	-
Starch	0.57	0.55	0.50	0.02	0.33	0.36	0.50	−0.34	0.02	−0.14	0.19	1.00	-	-
Ash	−0.38	−0.40	−0.83	0.29	−0.19	−0.11	−0.81	−0.05	−0.57	−0.51	−0.15	−0.54	1.00	-
Ca	−0.75	−0.73	−0.66	−0.16	−0.06	−0.06	−0.50	0.39	−0.21	−0.05	−0.22	−0.64	0.84	1.00
TP	0.32	0.28	−0.61	0.88	−0.34	−0.21	−0.84	−0.71	−0.71	−0.82	0.05	−0.19	0.64	0.16

DE, digestible energy; ME, metabolizable energy; GE, gross energy; CP, crude protein; AEE, acid-hydrolyzed ether extract; EE, ether extract; NDF, neutral detergent fiber; ADF, acid detergent fiber; TDF, total dietary fiber; IDF, insoluble dietary fiber; SDF, soluble dietary fiber; Ca, calcium; TP, total phosphorus.

1) The absolute values of data above 0.5 indicating the two chemical constituents are significantly correlated (p<0.05).

**Table 7:** Stepwise regression equations for digestible energy (DE) and metabolizable energy (ME) based upon the chemical characteristics of the 10 corn germ meal samples1)

Eq.	Liner regression equations	R^2^	RSD	p-value
1	DE (MJ/kg DM) = 23.49–0.62 ADF (%)	0.75	0.77	<0.01
2	DE (MJ/kg DM) = 22.28–0.46 ADF (%)–12.72 Ca (%)	0.90	0.51	<0.01
3	DE (MJ/kg DM) = 26.85–0.28 IDF (%)–17.79 Ca (%)	0.92	0.46	<0.01
4	ME (MJ/kg DM) = 22.14–0.57 ADF (%)	0.73	0.75	<0.01
5	ME (MJ/kg DM) = 21.05–0.43 ADF (%)–11.40 Ca (%)	0.87	0.56	<0.01
6	ME (MJ/kg DM) = 0.40+0.93 DE (MJ/kg DM)	0.98	0.22	<0.01

RSD, residual standard deviation; ADF, acid detergent fiber; Ca, calcium; IDF, insoluble dietary fiber; DM, dry matter.

1) Equations based on analyzed nutrient content expressed on a DM basis, n = 10.
